# Multiple imputation strategies for a bounded outcome variable in a competing risks analysis

**DOI:** 10.1002/sim.8879

**Published:** 2021-01-19

**Authors:** Elinor Curnow, Rachael A. Hughes, Kate Birnie, Michael J. Crowther, Margaret T. May, Kate Tilling

**Affiliations:** ^1^ Department of Statistics and Clinical Studies NHS Blood and Transplant Bristol UK; ^2^ Department of Population Health Sciences, Bristol Medical School University of Bristol Bristol UK; ^3^ Biostatistics Research Group, Department of Health Sciences University of Leicester, George Davies Centre Leicester UK; ^4^ MRC Integrative Epidemiology Unit University of Bristol Bristol UK; ^5^ Department of Medical Epidemiology and Biostatistics Karolinska Institutet Stockholm Sweden

**Keywords:** bounded data, competing risks, missing data, multiple imputation, predictive mean matching

## Abstract

In patient follow‐up studies, events of interest may take place between periodic clinical assessments and so the exact time of onset is not observed. Such events are known as “bounded” or “interval‐censored.” Methods for handling such events can be categorized as either (i) applying multiple imputation (MI) strategies or (ii) taking a full likelihood‐based (LB) approach. We focused on MI strategies, rather than LB methods, because of their flexibility. We evaluated MI strategies for bounded event times in a competing risks analysis, examining the extent to which interval boundaries, features of the data distribution and substantive analysis model are accounted for in the imputation model. Candidate imputation models were predictive mean matching (PMM); log‐normal regression with postimputation back‐transformation; normal regression with and without restrictions on the imputed values and Delord and Genin's method based on sampling from the cumulative incidence function. We used a simulation study to compare MI methods and one LB method when data were missing at random and missing not at random, also varying the proportion of missing data, and then applied the methods to a hematopoietic stem cell transplantation dataset. We found that cumulative incidence and median event time estimation were sensitive to model misspecification. In a competing risks analysis, we found that it is more important to account for features of the data distribution than to restrict imputed values based on interval boundaries or to ensure compatibility with the substantive analysis by sampling from the cumulative incidence function. We recommend MI by type 1 PMM.

## 1 INTRODUCTION

A purpose of patient follow‐up studies is to study the relationship between interventions or exposures and an event of interest. A frequent obstacle is that events are reported without an exact time of occurrence. This can be because the event takes place between periodic clinical assessments and so it is not possible to observe the exact time of onset. Studies in dentistry^1^ and human immunodeficiency virus (HIV)^2^ are typical examples from the literature. For example, Lesaffre and Komarek^1^ studied the time to tooth emergence in children. The time of emergence of a particular tooth occurs at some point between the last dental clinic at which no tooth is seen and the first clinic by which the tooth has emerged. Formally, such events are known as *bounded* or *interval‐censored*: for subject *i*, event times are known to lie in the interval (*L*_*i*_, *R*_*i*_], where *L*_*i*_ represents the last confirmed event‐free time and *R*_*i*_ represents the first time at which the event is known to have happened.

The problem of bounded data is a particular challenge in blood cancer studies. Long‐term remission from blood cancer is often only possible after hematopoietic stem cell (HSC) transplant. However, acute graft‐vs‐host disease (aGvHD) is one of a number of events that can increase patient mortality post‐transplant^3^ and time of onset of aGvHD is often not reported. The standard analysis of the cumulative incidence of aGvHD allows for the competing risks of graft failure and death prior to aGvHD.^4^ In some HSC studies, naive strategies have been used to handle missing aGvHD times, such as complete case analysis^5^ or substituting the mean of the observed times for all missing times,^4^ but this can lead to bias and under‐coverage.^6^, ^7^ Nikolajeva et al^8^ performed a logistic regression instead of the usual time‐to‐event analysis due to the large amount of missing aGvHD times in their study. This approach offers unbiased estimation of the incidence if there is complete follow‐up for all patients during the acute post‐transplant period (the first 100 days, approximately) but does lead to a loss of information, for example, about the median event time. If the bounded intervals are relatively narrow, Sun suggests substituting a single point from the interval (usually the mid‐point if there is no prior information about the part of the interval where the event is more likely to occur),^7^ but in most circumstances it is advisable to use a more rigorous approach. These can be broadly categorized as either (i) treating the event times as missing data and applying multiple imputation (MI) strategies or (ii) taking a full likelihood‐based (LB) approach. To our knowledge, these robust methods have not yet been applied to missing aGvHD times following HSC transplant.

Several different MI methods are available for handling bounded event times, some of which account for the skewed and strictly positive distribution of such times by using transformations, applying boundaries or sampling from the observed data. After MI, the completed datasets (with missing values replaced by imputed values) can be analyzed using standard statistical methods. Each dataset is separately analyzed and the multiple sets of results are combined using “Rubin's rules”.^9^ LB methods for interval‐censored competing risks data have also been developed: Hudgens et al^10^ derived a nonparametric maximum likelihood estimator (NPMLE) and later, a parametric estimator^11^ for the cumulative incidence function; Maathuis^12^, ^13^ computed the NPMLE of bivariate bounded data (their method can be adapted to estimate the cumulative incidence for univariate competing risks data); Mitra et al^14^ extended the parametric approach of Hudgens et al, allowing for missing event types, and Bakoyannis et al^15^ used a B‐spline sieve semi‐parametric maximum likelihood approach to estimate the cumulative incidence function for the class of generalized odds rate transformation models.

Here, we apply MI methods to data obtained from a registry of UK patients who received an HSC transplant. In our registry, the time of onset of aGvHD was frequently not reported although the times of competing events generally were. For cases of aGvHD with missing time of onset, the time of aGvHD was assumed to occur between day 0 (the transplant date) and day 100 (based on the standard clinical definition of aGvHD^16^) or the time of the patient's death, whichever occurred first. Since we had no other data to inform the interval boundaries, unless the patient died during the first 100 days, most spanned the interval (0, 100 days].

We focus on MI strategies, rather than LB methods, because our example deviates from the assumptions of the LB methods developed so far, in two ways: (i) our event times are a mixture of exact and interval‐censored times and (ii) our interval boundaries are the same for all interval‐censored event times and are wide relative to the observed event times. Furthermore, our desired analysis is non‐parametric estimation of the cumulative incidence function and Sun^7^ advises that non‐parametric LB methods should be avoided when intervals are wide. In contrast, the appeal of MI is its flexibility: after imputation, any desired complete data method may be used, a mixture of observed and missing data can be accommodated and additional data that are predictive of the missing times, but not required for the substantive analysis, can be used during the imputation step to inform the imputed times.

A key question of our paper is whether it is necessary to constrain the imputed times to lie within the censoring interval boundaries. Additionally, event times tend to be right‐skewed: this paper will investigate to what extent skewness should be accounted for in the imputation model. Previous studies have focused on skewed and bounded covariates; we will explore whether the same conclusions are drawn when imputing bounded outcomes. Another key question is that of compatibility between the imputation and analysis models. Previous studies have focused on standard, single‐cause, survival analysis. We will explore whether it is important that the imputation scheme incorporates the cumulative incidence function in order to achieve unbiased estimates in a competing risks framework. In a competing risks framework, the cumulative incidence function depends on all of the cause‐specific hazards, so the challenge of imputation increases dramatically.

Our approach is applicable to any study with bounded event times where the boundaries are the same for all subjects due to (i) clinical criteria or (ii) the design of the follow‐up reporting. Scenario (i) is common in pregnancy studies, for example. In a study of obesity and the risk of stillbirth,^17^ pregnancies that resulted in birth or stillbirth but with missing gestational age were excluded from analyses. Missing gestational age could have been treated as a bounded variable, with bounds of 20 and 42 weeks based on the study criteria. Scenario (ii) is common in studies with long intervals between follow‐up. For example, post‐transplant surgery was included as a risk factor in a corneal transplantation study.^18^ At 1, 2 and 5 years post‐transplant, hospitals were asked to report whether any surgery had been performed since the previous assessment but were not asked for the date of surgery. In this example, time of surgery was bounded by the previous and current assessment dates. Similarly, in a study of risk factors for self‐harm with and without suicidal intent,^19^ exposure data were collected annually or biannually from birth but participants were only asked at age 16/17 years whether they had harmed with suicidal intent at some point during their lifetime. Here the outcome of interest could have occurred at any point from birth to 16/17 years.

In this paper, we conduct an evaluation of MI strategies and one LB method for handling bounded outcomes. To emphasize the differences in our study from standard LB analysis of “interval‐censored” event times, we will use the term “bounded” to refer to the missing event times throughout this paper. We focus on the extent to which interval boundaries, the assumed distribution of imputed data and the substantive analysis model should be represented in the imputation model. In Section 2 we review the existing MI methodology in detail. In Section 3 we outline the possible missingness mechanisms. In Sections 4 and 5 we describe a simulation study comparing different MI strategies and one LB method and present its results. In Section 6 we apply the MI strategies considered to a real dataset. We conclude with general discussion in Section 7.

## 2 MI OF UNKNOWN EVENT TIMES

Several MI studies have focused on skewed and bounded covariates. Von Hippel^20^ found that attempts to normalize skewed variables via transformation led to biased results. He advises the use of untransformed variables when the purpose of the substantive analysis is to estimate associations, but the use of transformed variables when estimating percentiles. Rodwell et al^21^ found that restricting the range of the imputed values of skewed continuous covariates, either during the imputation step or post‐imputation, led to substantial bias. They concluded that it is best to impute on the raw scale with no range‐restrictions and no post‐imputation rounding. Lee and Carlin^22^ expanded on the work of von Hippel and Rodwell et al, comparing various transformations of nonnormal data to imputation on the raw scale and by predictive mean matching (PMM). In PMM, for each subject *i* with a missing value for variable *Y*, the following steps are performed: (i) calculate the predictive distance for all *h* subjects with an observed value for *Y*, (ii) identify a donor pool of subjects for which the predictive distance is minimized, (iii) randomly select a subject *d* from the donor pool, and (iv) replace the missing value of subject *i* with the observed value of subject *d*.

Morris et al^23^ describe three types of PMM, which differ in how the predictive distance is calculated. We shall briefly describe the calculation used in type 1 PMM and then explain how types 0 and 2 differ from type 1.

In type 1 PMM, the predictive distance is calculated as $\left|\beta^{*}z_{i}-\hat{\beta }z_{h}\right|$,

where $\hat{\beta }$ denote the estimates of regression coefficients ***β*** from a regression of *Y* on predictors **Z**, fitted to all *h* observed values of *Y*;

***β*** ^*^ denotes a random draw from the posterior distribution of ***β*** (or its Normal approximation)^24^; and

***z_i_*** and ***z***_***h***_ denote the values of ***Z*** for subjects *i* and *h*, respectively.

In type 0 the predictive distance is $\left|\hat{\beta }z_{i}-\hat{\beta }z_{h}\right|$ and in type 2 it is | ***β*** ^*^ ***z***_***i***_ − ***β*** ^*^ ***z***_***h***_|. Lee and Carlin found that type 1 PMM generally led to unbiased results. Several studies found that type 2 PMM led to under‐coverage.^21^, ^22^, ^23^

An assumption of MI is compatibility, or semi‐compatibility, between the imputation and substantive analysis models.^25^, ^26^ Compatibility requires that the imputation and substantive analysis models could be obtained by conditioning from the same joint model, while the weaker assumption of semi‐compatibility requires only that an imputation model can be made compatible by setting one or more parameters to zero. This allows for the inclusion of auxiliary variables^25^ in the imputation model to improve the imputation process even when these variables are not important in the substantive analysis. In survival analysis, it is well known that both the censoring indicator and a representation of the survivor function should be included in the imputation model.^25^, ^27^, ^28^ In models with missing covariates, White and Royston^28^ found that inclusion of the cumulative baseline hazard in the imputation model led to less biased results than if the survival time or log‐survival time were included and Bartlett et al^25^ found further improvement by using rejection sampling to exclude imputed values that were incompatible with a Cox regression model. Bartlett and Taylor^29^ extended Bartlett's approach to competing risks by considering cause‐specific hazard models. In models with missing outcome data, several authors have imputed bounded survival data by sampling directly from the survivor function. Hsu et al^30^ sampled from a Kaplan‐Meier estimate of the survivor function, while Pan^31^ sampled from a Cox model. Chen and Sun^32^ adapted Pan's method for an additive hazards model and Delord and Genin^33^ adapted Pan's method for non‐parametric and semi‐parametric cumulative incidence models in a competing risks framework.

## 3 MISSING DATA MECHANISMS

The mechanisms by which data are missing can be divided into three main categories.^34^ If the probability that data are missing is independent of the values of the observed and the missing data, then data are said to be missing completely at random (MCAR). If the probability that data are missing is independent of the missing data, conditional on the observed data, then data are said to be missing at random (MAR). Finally, if the probability that data are missing depends on the missing data itself, even after conditioning on the observed data, then data are said to be missing not at random (MNAR). If data are MNAR, knowledge of the missing data mechanism (MDM) is required for unbiased inference. Most MI methods are considered to be invalid if data are MNAR.^35^ We included MNAR mechanisms in our study to gauge in practice how large a bias would be introduced by using MI.

## 4 SIMULATION STUDY METHODS

The aim of the simulation study was to compare bias and precision of MI methods for handling bounded event times when estimating the cumulative incidence at 100 days post‐transplant and median time to aGvHD. In addition, we wanted to identify methods that were robust given a large proportion of missing data and/or imputation model misspecification.

### 4.1 Data‐generating mechanism

Similar to Grand et al,^36^ we generated event times directly from the cumulative incidence function using inverse transform sampling.^37^ For each subject *i*, the event type (1‐3) was determined with probability 0.65, 0.25, and 0.1 for aGvHD, graft failure prior to aGvHD and death prior to aGvHD, respectively. A *u*_*i*_ drawn from a uniform (0,1) distribution was used to determine each event time *t*_*i*_ such that:

$$u_{i}=p_{j}\left(t_{i}\right)\;\text{for event type}\;j=1,2,3,$$

where *p*_*j*_ *(t)* is the probability density function for a log‐normal distribution LN(*μ*_*j*_, *σ*_*j*_^2^) and *μ*_1_ = log(26), *σ*_1_ = log(2); *μ*_2_ = log(43), *σ*_2_ = log (2); *μ*_3_ = log(77), *σ*_3_ = log(4). The choice of distribution and parameter values were guided by the distribution of event times, measured in days, for the cohort in our registry. Each *t*_*i*_ was rounded up to the nearest whole number to increase computational speed and to reflect the real data.

We applied administrative censoring at 1 year post‐transplant for all subjects to reflect usual practice in transplant registry studies, that is, follow‐up until a fixed time‐point of clinical interest. We included one auxiliary variable, whether the subject received a double cord transplant (rather than a single cord), by sampling from a Bernoulli distribution with probability 0.45.

### 4.2 Estimands of interest

The estimands of interest were the cumulative incidence of aGvHD at 100 days post‐transplant and median time to aGvHD (the time by which 50% of patients had experienced aGvHD).

Since we were not imputing events (whether aGvHD, graft failure or death had occurred was known for every patient), we expected the estimated cumulative incidence to be unbiased in different scenarios. We included this estimand because we were interested in the precision of the estimate. We included the median as a measure of the shape of the cumulative incidence function. We expected the median to be a more sensitive gauge than the cumulative incidence to different MDM because the median depends on the (imputed) times of events, so different MI methods may lead to estimates with different biases and different precision.

The cumulative incidence function *F*{*t*} was estimated using the non‐parametric Aalen‐Johansen estimator^38^, ^39^ and its SE was estimated using the Greenwood‐style estimator described by Marubini and Valsecchi,^40^ as implemented in the “mstate” R (R Foundation for Statistical Computing, Vienna, Austria) package.^41^

We derived an estimator of the SE of the median using the delta method:

$$\mathrm{SE}\left[\hat{t}\left(50\right)\right]=\frac{1}{\hat{f}\{\hat{t}\left(50\right)\}}\mathrm{SE}\left[\hat{F}\left\{\hat{t}\left(50\right)\right\}\right].$$

The probability density function for the incidence of aGvHD at the median, $\hat{f}\left\{\hat{t}\left(50\right)\right\}$, was estimated by calculating a local gradient around the median, adapting the method suggested by Collett,^42^ such that:

$$\hat{f}\left\{\hat{t}\left(50\right)\right\}=\frac{\hat{F}\left\{\hat{u}\left(50\right)\right\}-\hat{F}\left\{\hat{l}\left(50\right)\right\}\;}{\hat{u}\left(50\right)-\hat{l}\left(50\right)},$$

where

$$\hat{u}\left(50\right)=\min\left\{t_{i},|,\hat{F}\left(t_{i}\right)\geq 0.5+\epsilon \right\}$$

and

$$\hat{l}\left(50\right)=\max\left\{t_{i},|,\hat{F}\left(t_{i}\right)\leq 0.5-\epsilon \right\},$$

for all *i* and small *ϵ* . We used *ϵ* = 0.01 throughout the simulation study.

### 4.3 Simulation size

One thousand datasets, each of size 500, were created using the data generating mechanism described above. The mean estimates for the 1000 datasets, with complete data, for the cumulative incidence at 100 days and median time of aGvHD are 63.11% (SE: 2.16%) and 44 days (SE: 3.61 days), respectively. Using the formula suggested by Burton et al^43^ with a type 1 error of 5%, 1000 datasets allows estimation of the cumulative incidence and median with a precision of 0.1% and 0.1 days, respectively.

### 4.4 Missing data mechanisms

Firstly, we considered a MCAR scenario. We set a random 10%, 30%, or 50% of all event times to missing and performed a complete case analysis (CCA) to confirm that this would result in unbiased estimates. We did not perform a full analysis when event times were MCAR because, in this scenario, missingness would be independent of event type. For consistency with findings for our registry cohort, we wanted the main analysis to be based on the assumption that times of graft failure and death were fully observed and only aGvHD times could be missing (ie, whether event times were missing or not depended on the type of event). Therefore, we focused on MAR and MNAR scenarios. In each dataset, 10%, 30%, or 50% of the aGvHD times (but not times of graft failure nor death) were set to missing using the following MDM:

- MAR:
*π*_*i*_ = 0.1, 0.3, or 0.5 for each subject *i* who experiences aGvHD.
*π_*i*_ = 0* for each subject *i* who experiences graft failure or death prior to aGvHD,
  where *π*_*i*_ denotes the probability that the event time for any subject *i* is missing.
- MNAR: The smallest 10%, 30%, or 50% of all aGvHD times were set to missing.

### 4.5 Performance measures

Performance measures of interest were:

- Standardized bias, defined as $\left(\frac{\bar{\hat{\beta }}-\beta }{\mathrm{SD}\left(\hat{\beta }\right)}\right)$, where $\bar{\hat{\beta }}=\sum_{k=1}^{1000}{\hat{\beta }}_{k}/1000$, ${\hat{\beta }}_{k}$ is the estimate within each simulation, $\mathrm{SD}\left(\hat{\beta }\right)$ is the SD of the ${\hat{\beta }}_{k}$ and *β* is the true value. We followed the advice of Burton et al^43^ that standardized bias can be more informative than bias or percentage bias because it accounts for the uncertainty of the estimate.
- Average model‐based SE, that is, the square root of $\sum_{k=1}^{1000}{\hat{\mathrm{SE}}}^{2}\left({\hat{\beta }}_{k}\right)\;/1000$ where $\hat{\mathrm{SE}}\left({\hat{\beta }}_{k}\right)$ is the estimated SE within each simulation.

### 4.6 Candidate methods for MI

The following imputation methods were considered. Each method was implemented using the “mice” R package^44^ with the default of five imputations. This number of imputations was deemed sufficient as there was only one incomplete variable. In order to test this assumption, methods were also implemented using 50 imputations for the MAR scenario.

- Normal imputation model with no restrictions on the imputed values (NORM), implemented using the method= “norm” option.
- Type 1 PMM imputation model with no restrictions on the imputed values (PMM), implemented using the method= “pmm” option.
- Log‐normal imputation model with postimputation back‐transformation (LOGNORM). The purpose of this scenario was to assess whether imputation of the median was improved when the normal distribution assumption was made more plausible as has been asserted.^20^ The natural logarithm of aGvHD time was imputed and the exponential of the imputed time was used in the analysis model.
- Normal regression with restrictions on the imputed values (RESNORM). The aim of this scenario was to test Rodwell's comment^21^ that restricting imputed values can increase rather than decrease bias. Within each imputation step, for each missing time value, a value was drawn and compared with the boundaries (0,100]. If this value was outside the boundaries, then a new value was drawn. This process was repeated until all imputed values were within the boundaries or until the process had been carried out 200 times. The cap on the number of times the process was repeated allowed for the possibility that the drawn value would never be within the boundary, because otherwise this would result in an endless loop through the process. The number of repeats was chosen to be large to minimize the number of imputed values outside the boundaries. As a sensitivity analysis, for the MAR scenario, this method was also implemented with the boundary comparison performed up to 500 times.

The event type (aGvHD, graft failure or death before aGvHD) was included in each imputation model for two reasons: (i) because it was part of the outcome of the substantive analysis and (ii) because it was the strongest predictor of missing event times. The number of cord blood (CB) units transplanted was included in each imputation model because, after event type, it was the strongest predictor of missing event times in the registry cohort. However, in the simulation study, it was not correlated with event times nor their probability of missingness.

We also considered models without the auxiliary variable (number of CB units) in the normal unrestricted and PMM imputation models so that we could directly compare bias and precision in models with and without an auxiliary variable, when the auxiliary variable was not predictive of aGvHD time. We referred to these models as NORMNOAUX and PMMNOAUX.

Finally, we considered the MI method proposed by Delord and Genin^33^ (referred to here as MICI) in which the non‐parametric cumulative incidence estimate is iteratively updated within the imputation step. In this method, values are sampled from the observed event times based on the current estimate of the cumulative incidence function, conditional on user‐defined boundaries. The sampling probability is determined by the current estimate of the cumulative incidence function. This method was implemented using an adaptation of the “MIICD” R package.^45^ We adapted the published package so that we could calculate the within‐imputation SE for each of our estimands. As a baseline for comparison, we performed a CCA.

In addition to the MI methods considered, we applied the B‐spline sieve semiparametric maximum likelihood approach of Bakoyannis et al (INTCCR) using the “intccr” R package,^46^ fitting a proportional subdistribution hazards (Fine and Gray^47^) model without covariates, because we wanted to compare MI methods with a LB method. We also wanted to test the authors' claim that the INTCCR method's performance was superior to that of Delord and Genin's approach.^46^ INTCCR only allows for half‐open intervals of the form (*L*_*i*_, *R*_*i*_], whereas exactly observed times require closed intervals. Therefore, for the exactly observed times in our simulated data, we set the left boundary to be the observed time minus 1 day and the right boundary to be the observed time. We combined graft failure and death into one event type as their package only allows for two event types. They used non‐parametric bootstrap sampling to estimate the SE of regression parameter estimates. We adapted the published package so that we could calculate the within‐imputation SE for each of our estimands using the same method.

The R code used to implement each method and generate the simulation results has been included in Appendix S1.

## 5 SIMULATION STUDY RESULTS

**FIGURE 1:**
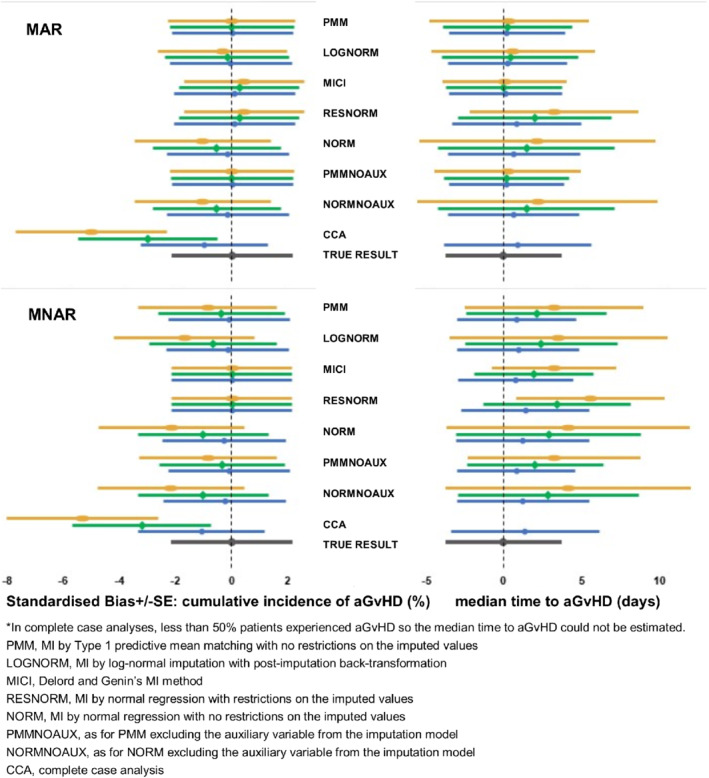
Standardized bias and average model‐based SE for cumulative incidence (%) of acute graft‐versus‐host disease (aGvHD) at 100 days post‐transplant and median time to aGvHD (days), with 10% (blue circle), 30% (green diamond) or 50% (yellow oval) missing times [Colour figure can be viewed at wileyonlinelibrary.com]

Simulation study results are illustrated in Figure 1 and tables of all results are included in Appendix S1. Figure 1 shows the standardized bias $\left(\frac{\bar{\hat{\beta }}-\beta }{\mathrm{SE}\left(\hat{\beta }\right)}\right)$ and average model‐based SE of the cumulative incidence of aGvHD at 100 days post‐transplant and median time to aGvHD for different imputation models, MDM and percentage of missing data. In Figure 1, methods for handling missing data are ranked in preferential order (most preferred at the top of each plot), based on bias and precision.

When event times were MCAR, that is, when there was an equal probability of any aGvHD, graft failure or death time being missing, CCA estimates of cumulative incidence and median were essentially unbiased as expected (standardized bias of the cumulative incidence of aGvHD at 100 days post‐transplant given 10%, 30%, and 50% missing times was: 0.04, 0.03, and 0.08, respectively, and standardized bias of the median time to aGvHD given 10%, 30%, and 50% missing times was: 0.20, 0.21, and 0.19, respectively).

In the estimation of the cumulative incidence, for both data MAR and MNAR, CCA greatly underestimated the cumulative incidence because times for outcomes other than aGvHD (death or graft failure prior to aGvHD) were fully complete and only cases of aGvHD were under‐represented. When data were MAR (given the event type), bias was small for all methods with the exception of CCA and methods based on unrestricted normal imputation (NORM and NORMNOAUX) (Figure 1). PMM performed particularly well, with negligible bias, even when 50% of event times were missing. NORM and NORMNOAUX under‐estimated the cumulative incidence at 100 days because the imputed times tended to be larger than with other imputation methods. Restricting the range of imputed values (RESNORM) reduced the absolute bias of the cumulative incidence estimates compared to NORM and NORMNOAUX. When the boundary comparison was performed in RESNORM a maximum of 200 times, a small number of imputed values were not within the boundaries. There was no change in the results when the boundary comparison was performed up to 500 times, but this did ensure that all imputed values were within the boundaries. As expected, SE for most methods increased with the volume of missing data, reflecting the additional uncertainty due to imputation. However, SE was under‐estimated for MICI and RESNORM methods; SE was the same or smaller than the full data SE in each scenario and was similar regardless of the volume of missing data. Results for the LB method INTCCR were very similar to those for the MICI method; SE was under‐estimated with this method too, although not to the same extent. When data were MNAR, all methods under‐estimated the cumulative incidence except MICI, RESNORM and INTCCR methods. The lack of bias in the results for these methods can be explained by their tendency to left‐skew the cumulative incidence function as reported for the MAR scenario. As a check of this statement, the MNAR analysis was repeated for MICI, RESNORM, and INTCCR methods, setting largest times to missing instead of smallest times. The cumulative incidence was over‐estimated as expected, by approximately 0.8 SDs for each method and each percentage missing data.

In the estimation of the median, in CCA for both MAR and MNAR scenarios, less than 50% of all patients experienced aGvHD when more than 10% aGvHD times were missing and so the median time to aGvHD could not be estimated. When data were MAR, bias was small for MICI, PMM, and INTCCR methods. Overall the size of the standardized bias was larger for median than for cumulative incidence estimation. NORM and NORMNOAUX over‐estimated the median and restricting the range of imputed values did not improve median estimation. When data were MNAR, all methods over‐estimated the median, with bias greater than 0.5 SD for all methods, even when only 10% of aGvHD times were missing. For both cumulative incidence and median estimation, including the auxiliary variable (number of CB units) in the PMM and NORM imputation models did not reduce the bias and resulted in larger SE.

The same pattern of results was seen when the number of imputations was increased to 50 (see Appendix  S1).

## 6 APPLICATION TO REAL DATA: aGvHD IN HSC TRANSPLANT PATIENTS

Between 1996 and 2015, 432 first HSC transplants from unrelated donors were performed using CB provided by the UK National Health Service (NHS) Cord Blood Bank^48^ (CBB). The patients included both adult and pediatric (aged 16 years or less) patients, treated for malignant and nonmalignant blood disorders. Of all 432 patients, 241 experienced aGvHD and 57 (24%) of these did not have a date of onset reported. The observed times of onset were between 5 and 119 days posttransplant, which is slightly outside the standard definition of the “acute” period. Thirty patients experienced graft failure and 81 patients died prior to the onset of aGvHD. Eighty patients were alive with a functioning graft and aGvHD‐free at last follow‐up.

Cases with missing aGvHD times were more likely to be double (vs single) CB unit transplants (63% double CB unit transplants where aGvHD time was missing vs 41% where aGvHD time was reported, *P* = 0.004). In order to achieve sufficiently high HSC doses per kg body weight, adults are more likely to receive double CB unit transplants and on average patients with missing aGvHD times were older (mean, SD age at transplant 33, 25 years vs 25, 22 years where aGvHD time was reported, *P* = 0.039). These results suggest that aGvHD times were not MCAR.

Previous studies have found an association between time of onset of aGvHD and both aGvHD grade and overall survival: Lee et al^49^ found that early onset aGvHD (onset prior to myeloid engraftment) was associated with more severe grades of aGvHD than late onset aGvHD; Omer et al^50^ found overall survival was worse in patients with persistent late aGvHD (onset after 100 days). We found no evidence of a difference in grade (severity) of aGvHD (*P* = 0.692) nor overall survival (*P* = 0.296) between cases with and without aGvHD time reported, which suggests that aGvHD times in our study were MAR rather than MNAR.

We applied all methods considered in the simulation study to the NHS CBB dataset. As well as the event experienced (aGvHD, graft failure or death prior to aGvHD), we included patient, donor and transplant characteristics predictive of aGvHD time in its imputation model (see Appendix S1 for further details). Several of these characteristics were also incomplete. Missing values were imputed using standard methods: continuous variables using Type 1 PMM, binary variables using logistic regression and categorical variables using multinomial regression.

It was of interest to see whether the methods considered for imputation of aGvHD time performed well in a setting where multiple variables were imputed. Multivariate imputation was performed by fully conditional specification (FCS) for all imputation methods considered in the simulation study apart from Delord and Genin's method, which is a univariate imputation method. In the FCS method^51^ (also referred to as MI by chained equations^44^), difficulties with specifying a joint distribution for the missing data in step 1 of the MI process are overcome by creating a series of univariate imputation models (one for each incomplete variable) which are sampled from in turn, conditional on all other observed and imputed data (see Appendix S1 for further details). In our example, the number of imputations equalled 24, the percentage of missing aGvHD times.^24^

As in the simulation study, we considered models without the auxiliary variables (ie, excluding all patient, donor, and transplant characteristics) in the normal unrestricted and PMM imputation models. In contrast to the simulation study (in which the auxiliary variable was not predictive of aGvHD time), the purpose this time was to compare bias and precision in models with and without auxiliary variables when these were predictive of aGvHD time.

Estimates and SE of the cumulative incidence at 100 days post‐transplant and median time of aGvHD for the NHS CBB cohort are shown in Table 1. Results are consistent with those from the simulation study: PMM, LOGNORM, MICI, and RESNORM imputation led to similar estimates for the cumulative incidence at 100 days and median time to aGvHD of (average for the four methods) 55% (SE: 2%) and 74 days (SE: 12 days), respectively. Estimates from methods based on unrestricted normal imputation models (NORM, NORMNOAUX) were smaller than for other methods for the cumulative incidence and larger for the median time to aGvHD, with very large SE for the median. For median but not cumulative incidence estimation, including the auxiliary variables in the PMM and NORM imputation models resulted in smaller SE than for models excluding the auxiliary variables (NORMNOAUX, PMMNOAUX).

**TABLE 1:** Estimate and SE of the cumulative incidence at 100 days post‐transplant and median time of aGvHD for the UK National Health Service (NHS) Cord Blood Bank (CBB) cohort for various imputation methods

Estimand	Cumulative incidence at 100 days (%)		Median (days)	
Imputation method	Estimate	SE	Estimate	SE
PMM	54.84	2.45	75.46	11.24
LOGNORM	55.17	2.45	69.39	14.07
MICI	55.70	2.41	69.63	14.29
RESNORM	54.03	2.48	83.04	8.07
NORM	51.04	2.58	95.73	74.07
PMMNOAUX	55.46	2.45	72.25	12.30
NORMNOAUX	49.55	2.57	144.43	152.08
CCA^a^	48.92	2.61	n/a	n/a

Abbreviations: CCA, complete case analysis; LOGNORM, FCS MI by log‐normal imputation with post‐imputation back‐transformation; MICI, Delord and Genin's MI method; NORM, FCS MI by normal regression with no restrictions on the imputed values; NORMNOAUX, as for NORM excluding the auxiliary variables from the imputation model; PMM, FCS MI by Type 1 predictive mean matching with no restrictions on the imputed values; PMMNOAUX, as for PMM excluding the auxiliary variables from the imputation model; RESNORM, FCS MI by normal regression with restrictions on the imputed values.

a In complete case analyses, less than 50% patients experienced aGvHD so the median time to aGvHD could not be estimated.

## 7 DISCUSSION

We used a simulation study to evaluate the extent to which interval boundaries, the data distribution and the substantive analysis model should be represented in the imputation model for bounded event times. We compared methods in two different missing data scenarios, while also varying the percentage of missing data. We found that cumulative incidence and median event time estimation were sensitive to model misspecification.

We found that sampling from a set of observed times (PMM, Delord and Genin), without reliance on a specific parametric distribution, resulted in broadly unbiased estimates. In PMM, missing values are replaced by sampling at random from a donor pool of subjects (with observed values) who are “similar” to the subject with missing data. In Delord and Genin's method, sampling is from the set of observed times that lie within the censored interval boundaries, where the sampling probability is determined by the current estimate of the cumulative incidence function. Delord and Genin's method resulted in the least biased estimates of the median, which suggests that compatibility with the substantive analysis model is important in median estimation. However, SE was under‐estimated using this method. It also has the drawback of being a univariate imputation method. Therefore, we recommend using type 1 PMM when imputing bounded event times.

Restricting the range of imputed values generally reduced the bias for cumulative incidence estimation, though not for median estimation. SE was under‐estimated using this method. The method of Delord and Genin and the restricted normal both truncate values drawn during the imputation step and this seems to lead to under‐estimates of the SE. For this reason, restricted range methods are not recommended. We also do not completely agree with von Hippel's advice to transform variables with a skewed distribution when estimating percentiles (in our case, via log‐transformation); although estimates were improved in comparison with an untransformed normal imputation model, log‐transformation resulted in some bias.

Contrary to other publications in this field,^20^, ^21^ in our simulation study, imputing on the raw scale led to bias. However, this may be due to imputation model misspecification, more specifically, due to the constant variance assumption of the normal imputation model.^24^ In our simulation study, guided by the distribution of event times for each event type in our registry cohort, aGvHD and graft failure times were generated with a variance of (log (2))^2^ whereas death times were generated with a variance of (log (4))^2^. Including all event types in the imputation model resulted in a model with estimated variance greater than desired, which explains the tendency of this method toward larger times compared with other imputation methods. We corrected this by limiting the normal imputation model to the subset of patients who experienced aGvHD, resulting in estimates with negligible bias even when 50% data were missing (see Appendix S1). We recommend exploring the distribution of event times for different sub‐groups of subjects prior to imputation. In theory, for any subgroup for which the distribution of event times is substantially different, a separate imputation model could be fit. However, this may not be practical in studies with large numbers of variables included in the imputation models or many incomplete variables to be imputed. Therefore, overall, we recommend MI by FCS with type 1 PMM, because of its performance and flexibility.

There was no association between the auxiliary variable and the event time in the simulation study. This may explain why inclusion of the auxiliary variable in the imputation model did not reduce bias and increased SE.^52^ Conversely, in the real data example, inclusion of auxiliary variables that were predictors of event times reduced the SE of the median estimate. Overall, we advise using an inclusive strategy^52^ to reduce bias, increase efficiency and make the MAR assumption more plausible, by including in the imputation model all variables, including outcome variables, required for the substantive analysis as well as variables predictive of the incomplete variable or its missingness. When data were MAR, imputation led to broadly unbiased estimates. Generally, MI techniques are not recommended for data MNAR and we found that imputation resulted in biased estimates when data were MNAR even when only a small percentage of data were missing.

We did not find any advantage in using a LB method rather than MI to handle missing event times. MI offers many advantages, notably the option to include auxiliary data during imputation, flexibility when choosing the analysis model and accommodation of a mixture of exactly observed and missing times. As far as we know, there is only one standard software application of LB methods that allows calculation of the non‐parametric estimate of the cumulative incidence function. This is the package “MLEcens”^13^ available in R. However, it only allows for two competing events and does not provide SE estimation. In contrast, MI can be easily implemented in many statistical packages including SAS (SAS Institute Inc., Cary, North Carolina), Stata (StataCorp, College Station, Texas) and R software.

It is particularly important to handle missing aGvHD times appropriately because aGvHD is a commonly reported outcome in HSC studies. Little and Rubin^53^ note that a complete case analysis will always be biased if missingness depends on the substantive analysis outcome. Our study results support this statement. However complete case analysis and naïve imputation methods are frequently used to handle missing times in HSC studies.^4^, ^8^ We recommend MI by type 1 PMM to impute missing aGvHD times when analysis includes estimation of the cumulative incidence and median event time. It is straightforward to implement PMM in many software packages although it should be noted that SAS software only implements type 2 PMM,^23^ which has inferior performance to type 1 PMM.^21^, ^22^

Our results can be more widely applied to any analysis with missing event times, not just studies in transplantation. They apply particularly when there is reason to believe that event times are MAR, when the interval boundaries are uninformative, when there is a mixture of observed and missing times and when there are competing events.

This study considered a simple analysis model (non‐parametric estimation of cumulative incidence function). Further work is required to determine whether results also apply to models involving covariates, by, for example, examining subdistribution hazard ratios from a Fine and Gray model, the hazard ratio for aGvHD in a time‐dependent Cox regression model of overall survival or the transition intensities in a multi‐state model including multiple intermediate events.

## CONFLICT OF INTERESTS

The authors declare that there is no conflict of interest.

## Supporting information


**Appendix S1**: supporting information

## Data Availability

The data that support the findings of this study are available on request from the corresponding author. The data are not publicly available due to privacy restrictions.
