# Supplementary material for: Multiple imputation strategies for a bounded outcome variable in a competing risks analysis
Source: Stat Med. 2021 Jan 19;40(8):1917–29. doi: 10.1002/sim.8879 (PMC8611803; doi:10.1002/sim.8879)
Supplement: Supplementary file 1 — Appendix S1: supporting information [file SIM-40-1917-s001.docx]

**Multiple imputation strategies for a bounded outcome variable in a competing risks analysis**

E. Curnow^1,2^, R. A. Hughes^2^, K. Birnie^2^, M. J. Crowther^3^, M. T. May^2^, K. Tilling^2^

^1^ Department of Statistics and Clinical Studies, NHS Blood and Transplant, Bristol, UK

^2^ Department of Population Health Sciences, Bristol Medical School, University of Bristol, Bristol, UK

^3^ Department of Health Sciences, University of Leicester, Centre for Medicine, Leicester, UK

**Supplementary Material**

**Table 1. Standardised bias (StdBias) and average model-based SE (AvModSE) for (A) MCAR, (B) MAR, (C) smallest values MNAR. Complete data estimates of the cumulative incidence at 100 days post-transplant and median time to aGvHD were 63.11% (SE: 2.16%) and 44 days (SE: 3.61 days) respectively. Number of imputations = 5.**

| **Missing data mechanism** | **Estimand** | **Cumulative incidence** | | | | | | **Median** | | | | | |
| --- | --- | --- | --- | --- | --- | --- | --- | --- | --- | --- | --- | --- | --- |
|  | **Proportion of bounded times** | **10%** | | **30%** | | **50%** | | **10%** | | **30%** | | **50%** | |
|  | **Method** | **Std**  **Bias** | **AvModSE** | **StdBias** | **AvModSE** | **StdBias** | **AvMod**  **SE** | **StdBias** | **AvMod**  **SE** | **StdBias** | **AvMod**  **SE** | **StdBias** | **AvMod**  **SE** |
| **MCAR** | CCA | 0.04 | 2.27 | 0.03 | 2.58 | 0.08 | 3.04 | 0.20 | 3.78 | 0.21 | 4.39 | 0.19 | 5.53 |
| **MAR** | PMM | 0.02 | 2.17 | 0.01 | 2.20 | -0.01 | 2.27 | 0.21 | 3.73 | 0.26 | 4.17 | 0.34 | 5.02 |
|  | PMMNOAUX | 0.02 | 2.17 | 0.01 | 2.19 | 0.01 | 2.22 | 0.20 | 3.71 | 0.22 | 4.03 | 0.24 | 4.60 |
|  | LOGNORM | -0.03 | 2.17 | -0.16 | 2.22 | -0.33 | 2.29 | 0.27 | 3.82 | 0.44 | 4.37 | 0.62 | 5.17 |
|  | MICI | 0.10 | 2.15 | 0.27 | 2.15 | 0.44 | 2.14 | 0.13 | 3.63 | 0.03 | 3.71 | 0.06 | 3.89 |
|  | RESNORM | 0.10 | 2.15 | 0.27 | 2.15 | 0.44 | 2.14 | 0.86 | 4.16 | 2.01 | 4.92 | 3.25 | 5.31 |
|  | RESNORM500 | 0.10 | 2.15 | 0.27 | 2.15 | 0.44 | 2.14 | 0.86 | 4.16 | 2.01 | 4.92 | 3.25 | 5.31 |
|  | NORM | -0.14 | 2.19 | -0.53 | 2.28 | -1.04 | 2.43 | 0.67 | 4.25 | 1.48 | 5.69 | 2.16 | 7.48 |
|  | NORMNOAUX | -0.14 | 2.19 | -0.53 | 2.28 | -1.04 | 2.44 | 0.67 | 4.23 | 1.48 | 5.67 | 2.18 | 7.59 |
|  | NORMSUBGRP | 0.02 | 2.16 | 0.02 | 2.18 | 0.02 | 2.20 | 0.20 | 3.70 | 0.20 | 3.94 | 0.19 | 4.13 |
|  | CCA* | -0.98 | 2.26 | -2.99 | 2.48 | -5.02 | 2.71 | 0.92 | 4.74 | n/a | n/a | n/a | n/a |
|  | INTCCR | 0.05 | 2.15 | 0.25 | 2.17 | 0.41 | 2.20 | 0.17 | 4.10 | 0.05 | 4.16 | -0.04 | 4.42 |
| **MNAR** | PMM | -0.07 | 2.17 | -0.36 | 2.25 | -0.85 | 2.48 | 0.84 | 3.81 | 2.12 | 4.51 | 3.25 | 5.62 |
|  | PMMNOAUX | -0.07 | 2.17 | -0.34 | 2.23 | -0.83 | 2.44 | 0.84 | 3.80 | 2.04 | 4.38 | 3.25 | 5.44 |
|  | LOGNORM | -0.13 | 2.18 | -0.66 | 2.27 | -1.68 | 2.51 | 0.95 | 3.92 | 2.43 | 4.90 | 3.54 | 6.92 |
|  | MICI | 0.02 | 2.15 | 0.02 | 2.15 | 0.02 | 2.15 | 0.78 | 3.71 | 1.97 | 3.82 | 3.25 | 3.88 |
|  | RESNORM | 0.02 | 2.15 | 0.02 | 2.15 | 0.02 | 2.15 | 1.41 | 4.14 | 3.43 | 4.75 | 5.56 | 4.66 |
|  | NORM | -0.25 | 2.19 | -1.01 | 2.33 | -2.14 | 2.59 | 1.25 | 4.30 | 2.90 | 5.92 | 4.14 | 7.71 |
|  | NORMNOAUX | -0.24 | 2.19 | -1.01 | 2.33 | -2.16 | 2.61 | 1.25 | 4.25 | 2.88 | 5.81 | 4.16 | 7.79 |
|  | CCA* | -1.06 | 2.26 | -3.19 | 2.48 | -5.31 | 2.71 | 1.40 | 4.77 | n/a | n/a | n/a | n/a |
|  | INTCCR | -0.03 | 2.15 | 0.00 | 2.17 | -0.01 | 2.18 | 0.80 | 4.15 | 2.10 | 4.19 | 3.56 | 4.28 |

*In these complete case analyses, less than 50% patients experienced aGvHD so the median time to aGvHD could not be estimated.

PMM, MI by Type 1 predictive mean matching with no restrictions on the imputed values

PMMNOAUX, as for PMM excluding the auxiliary variable from the imputation model

LOGNORM, MI by log-normal imputation with post-imputation back-transformation

MICI, Delord and Genin’s (2015) MI method

RESNORM, MI by normal regression with restrictions on the imputed values and boundary comparison performed up to 200 times

RESNORM500, MI by normal regression with restrictions on the imputed values and boundary comparison performed up to 500 times

NORM, MI by normal regression with no restrictions on the imputed values

NORMNOAUX, as for NORM excluding the auxiliary variable from the imputation model

NORMSUBGRP, as for NORM with the imputation model limited to cases of aGvHD

CCA, complete case analysis

INTCCR, B-spline sieve maximum likelihood method of Bakoyannis *et al.* (2017).

All MI methods included the auxiliary variable except PMMNOAUX and NORMNOAUX.

**Table 2. Standardised bias (StdBias) and average model-based SE (AvModSE) for MAR. Complete data estimates of the cumulative incidence at 100 days post-transplant and median time to aGvHD were 63.11% (SE: 2.16%) and 44 days (SE: 3.61 days) respectively. Number of imputations = 50.**

| **Missing data mechanism** | **Estimand** | **Cumulative incidence** | | | | | | **Median** | | | | | |
| --- | --- | --- | --- | --- | --- | --- | --- | --- | --- | --- | --- | --- | --- |
|  | **Proportion of bounded times** | **10%** | | **30%** | | **50%** | | **10%** | | **30%** | | **50%** | |
|  | **Method** | **Std**  **Bias** | **AvModSE** | **StdBias** | **AvModSE** | **StdBias** | **AvModSE** | **StdBias** | **AvModSE** | **StdBias** | **AvModSE** | **StdBias** | **AvModSE** |
| **MAR** | PMM | 0.02 | 2.16 | 0.01 | 2.19 | -0.01 | 2.25 | 0.21 | 3.70 | 0.26 | 4.08 | 0.34 | 4.88 |
|  | LOGNORM | -0.03 | 2.17 | -0.17 | 2.21 | -0.34 | 2.27 | 0.27 | 3.79 | 0.44 | 4.30 | 0.63 | 5.04 |
|  | MICI | 0.10 | 2.15 | 0.27 | 2.15 | 0.44 | 2.14 | 0.13 | 3.61 | 0.02 | 3.68 | 0.06 | 3.83 |
|  | RESNORM | 0.10 | 2.15 | 0.27 | 2.15 | 0.44 | 2.14 | 0.87 | 4.14 | 2.07 | 4.85 | 3.32 | 5.20 |
|  | NORM | -0.14 | 2.18 | -0.54 | 2.26 | -1.05 | 2.39 | 0.68 | 4.21 | 1.50 | 5.59 | 2.22 | 7.29 |
|  | CCA* | -0.98 | 2.26 | -2.99 | 2.48 | -5.02 | 2.71 | 0.92 | 4.74 | n/a | n/a | n/a | n/a |

*In complete case analyses, less than 50% patients experienced aGvHD so the median time to aGvHD could not be estimated.

PMM, MI by Type 1 predictive mean matching with no restrictions on the imputed values

LOGNORM, MI by log-normal imputation with post-imputation back-transformation

MICI, Delord and Genin’s (2015) MI method

RESNORM, MI by normal regression with restrictions on the imputed values

NORM, MI by normal regression with no restrictions on the imputed values

CCA, complete case analysis.

All MI methods included the auxiliary variable.

**Multiple imputation by fully conditional specification**^1^

For each incomplete variable *X*, an imputation model *f(x* | ***x****_-_, θ)* and non-informative prior *p(θ)* for the model parameter *θ* are specified. ***X****_-_* represents the set of variables excluding *X*, *x^obs^* and *x^mis^* represents the observed and missing data respectively for variable *X* and * denotes the last drawn value for any element of ***X****_-_*. Each imputation consists of *T* iterations.

First, arbitrary starting values are assigned to all missing data.

Then, in iteration *t =* 2,…*T*, for each variable in turn, *θ^(t)^* is drawn from a distribution proportional to *p(θ)p(x^obs^* | ***x****_-_^*^, θ)*. Missing values *x^mis(t)^* are then drawn from *f(x* | ***x****_-_^*^, θ^(t)^)*. Values from the *T^th^* iteration are retained as the imputed dataset. The process is repeated *M* times to create *M* imputed datasets.

**Patient, donor and transplant characteristics included in the real-data application (with percentage missing data)**

Number of CB units transplanted (0%); patient age (0%); disease type (0%) and disease status (35%) (relapse, remission, other) at transplant; pre-transplant radiotherapy/chemotherapy regimen (4%) (intensive or reduced intensity); gender (1%) and cytomegalovirus-positive (CMV+) (12%) match between donor(s) and recipient; number of human leucocyte antigen (HLA) mismatches (13%) between donor(s) and recipient; dose at infusion (24%) (measured by the total nucleated cell count infused x 10^7^/kg patient weight); year of transplant (0%); time to myeloid engraftment (1%) (defined as three consecutive days with an absolute neutrophil count of at least 0.5 x 10^9^/l); indication of myeloid engraftment (vs. graft failure or death prior to engraftment) (0%). In order to focus on just one missing event time, seven patients missing time of graft failure or myeloid engraftment were excluded from the analysis.

**R Code to generate simulation results**

1. **Required libraries**

library(survival)

library(mstate)

library(MIICD)

library(mice)

library(intccr)

library(doParallel)

1. **Data Generating Mechanism**

#Create simulation datasets based on average of 65% GvHD, 10% graft failures, 25% deaths

sample500=data.frame()

for (i in 1:1000)

{

sample=rbind(cbind.data.frame(agvhd_status_nocens=1,runif=runif(325)),

cbind.data.frame(agvhd_status_nocens=2,runif=runif(50)),

cbind.data.frame(agvhd_status_nocens=3,runif=runif(125)))

#define additional runif for ordering

sample$rorder=runif(500)

sample500=rbind.data.frame(sample500,sample)

}

#Now simulate times and round times to match observed data

sample500$agvhd_time=ifelse(sample500$agvhd_status_nocens == 1,

ceiling(exp(qnorm(p=sample500$runif,mean=3.26,sd=0.71))),

ifelse(sample500$agvhd_status_nocens == 2,

ceiling(exp(qnorm(p=sample500$runif,mean=3.76,sd=0.66))),

ceiling(exp(qnorm(p=sample500$runif,mean=4.34,sd=1.3)))))

#Censoring event times at greater than one year

sample500$agvhd_status=ifelse(sample500$agvhd_time<=365,sample500$agvhd_status_nocens,0)

sample500$agvhd_time[sample500$agvhd_status==0]=365

#also add one covariate - number of cords received - with 45% double cord

sample500$doublecord=rbinom(500000,1,0.45)

#Indicator of agvhd

sample500$agvhd=ifelse(sample500$agvhd_status==1,1,0)

#Order all obs randomly before adding a sample number for each of the 1000 datasets

sample500=sample500[order(sample500$rorder),]

sample500$sampno=rep(1:1000, 500)

1. **Functions to apply the missing data mechanisms used in the study**
   1. MCAR

MCAR = function(dset,time,percent)

# dset = dataset, time = event time, percent = percent missing data as a decimal

# e.g.

# sample1$time.miss=MCAR(dset=sample1,time=sample1$agvhd_time,percent=0.1)

{

time.miss=c()

for (j in 1: nrow(dset))

{time.miss[j]=ifelse(rbinom(1,1,percent)==1,NA,time[j])}

return(time.miss)

}

- 1. MAR with missingness dependent on event type

MAR = function(dset, time, percent, event)

# dset = dataset, time = event time, percent = percent missing data as a decimal

#event = indicator variable: 1 if event type of interest and 0 otherwise

# e.g.

# sample1$time.miss=MAR(dset=sample1,time=sample1$agvhd_time,percent=0.1,

#event=sample1$agvhd)

{

time.miss=c()

for (j in 1: nrow(dset))

{time.miss[j]=ifelse(rbinom(1,1,percent)==1 & event[j]==1,NA,time[j])}

return(time.miss)

}

- 1. MNAR with smallest event times missing

MNAR = function(dset, time, percent, event)

# dset = dataset, time = event time, percent = percent missing data as a decimal

#event = indicator variable: 1 if event type of interest and 0 otherwise

# e.g.

# sample1$time.miss=MNAR(dset=sample1,time=sample1$agvhd_time,percent=0.1,

#event=sample1$agvhd)

{

dset.tmp=data.frame(cbind(event.tmp=event, time.tmp=time,id=c(1:nrow(dset))))

dset.tmp=dset.tmp[order(-dset.tmp$event.tmp, dset.tmp$time.tmp),]

dset.tmp$time.miss=dset.tmp$time

dset.tmp$time.miss[1:round(percent*nrow(dset.tmp[dset.tmp$event.tmp==1,]))]=NA

dset.tmp=dset.tmp[order(dset.tmp$id),]

return(dset.tmp$time.miss)

}

1. **Functions to apply the multiple imputation methods used in simulation study**

In each function, time = event time with missing times generated as per one of the missing data mechanisms, type = event type indicator, var = covariate and nimp = number of imputations *e.g.* imp=NORM(time=sample1$time.miss, type=sample1$agvhd_status, var=sample1$doublecord, nimp=5)

- 1. Normal imputation model with no restrictions on the imputed values

NORM = function(time, type, var, nimp)

{

midata=data.frame(time=time, type=type , var)

imp=mice(midata, m=nimp,defaultMethod=c('norm','logreg','polyreg'),print=FALSE)

return(imp)

}

NORMNOAUX= function(time, type, nimp) #without covariate

{

midata=data.frame(time=time, type=type)

imp=mice(midata, m=nimp,defaultMethod=c('norm','logreg','polyreg'),print=FALSE)

return(imp)

}

- 1. Type 1 PMM imputation model with no restrictions on the imputed values

PMM = function(time, type, var, nimp)

{

midata=data.frame(time=time, type=type , var)

imp=mice(midata,m=nimp,print=FALSE)

return(imp)

}

PMMNOAUX = function(time, type, nimp) #without covariate

{

midata=data.frame(time=time, type=type)

imp=mice(midata,m=nimp,print=FALSE)

return(imp)

}

- 1. Log-normal imputation model with post-imputation back-transformation

LOGNORM = function(time, type, var, nimp)

{

midata=data.frame(time=log(time), type=type , var)

imp=mice(midata, m=nimp,defaultMethod=c('norm','logreg','polyreg'),print=FALSE)

imp[["imp"]]$time=exp(imp[["imp"]]$time)

return(imp)

}

- 1. Normal regression with restrictions on the imputed values (RESNORM).

# Adapt normal imputation method

mice.impute.norm3 = function (y, ry, x,...)

{

valid_vals <- rep(NA, length.out = sum(!ry))

# Counter to avoid endless loop

cntr = 0

repeat{

vals = mice.impute.norm(y, ry, x, ...)

#Compare with boundaries

correct = vals > 0 & vals <= 100

if (all(!is.na(valid_vals) | correct)){

valid_vals[correct] = vals[correct]

break

#stop if all values within boundaries

}

else if (any(is.na(valid_vals) & correct)){

valid_vals[correct] = vals[correct]

}

cntr = cntr + 1

if (cntr > 200){

if (all(is.na(valid_vals))){

valid_vals = vals

}

else{

valid_vals[is.na(valid_vals)] = vals[is.na(valid_vals)]

}

break

}

}

return(valid_vals)

}

RESNORM = function(time, type, var, nimp)

{

midata=data.frame(time=time, type=type , var)

imp=mice(midata, m=nimp, method=c(time = "norm3"),print=FALSE)

return(imp)

}

- 1. MI method proposed by Delord and Genin

#Adaptation of the MI.ci function and associated functions from the ‘MIICD’ R #package

preproc.crreg_2 <- function( data = data , m = m , trans = trans , status = status , cens.code = cens.code ){

rownames(data)<-seq_len(nrow(data))

I <- data[,'left'] != data[,'right' ] & data[,'right'] != Inf & data[,status] != cens.code

data2<-data[I,]

dim(data2)

dataE<-data[!rownames(data)%in%rownames(data2),]

or<-order(c(as.numeric(rownames(data2)),as.numeric(rownames(dataE))))

data1<-t(apply( dataE , 1 , function(x) as.numeric(rep(x['left'] , m ))))

dim(data1)[1]+dim(data2)[1]

return( list( data2 = data2 , data1 = data1 , or = or, I = I ) )

}

get_z2<-function(data){

s1<-sapply( 1:ncol(data) , function(y) sapply(1:nrow(data), function(x) length(unlist(data[x,y]))) )

data[s1==0]<-0

s2<-sapply( 1:ncol(data) , function(y) sapply(1:nrow(data), function(x) unlist(data[x,y])))

return(s2)}

MI.ci1<-function( m , status , trans , data , conf.int = TRUE , cens.code , alpha = 0.05 , ntimes = NULL ){

if( !is.numeric(m) ) stop('m must be an integer')

if( !is.data.frame(data) ) stop('data must be a data.frame')

if( !is.logical(conf.int) ) stop('conf.int must be logical')

if( alpha <= 0 | alpha >= 1 ) stop('alpha must be in ] 0 , 1 [')

cl<-match.call()

#Use interval censored data and generate k sets of imputed data

sets<-sapply( 1:m , get.set2 , data )

#Get and sort single times at wich the cumulative incidence will be estimated

times<-as.vector(sets)

length(times)

###

r2 <- as.character( rep( data[ , status ] , m ) )

r2<-factor(r2,levels=unique(c(cens.code,unique(r2))))

r1 <- as.character( data[ , status ] )

r1<-factor(r1,levels=unique(c(cens.code,unique(r1))))

fitCI <- survfit( Surv( time = times , event = r2 , type = "mstate" ) ~ 1 , weights = rep( 1 / m , length(times) ) ,

conf.type = 'none' )

w <- which( fitCI$states == trans )

pr <- fitCI$pstate[ , w ]

sd <- fitCI$std.err[ , w ]

t0 <- fitCI$time

#get estimated of cumulative incidence and confidence intervals

if(! is.null(ntimes) ){

t1 <- seq( from = range(t0)[1] , to=range(t0)[2] , length = ntimes )

sd_at_times<-sapply( 1 , get_values_at_times2 , values = list(sd) , times = list(t0) , at = t1 , list = T )

sd_at_times <- get_z2( sd_at_times )

ci_at_times<-sapply( 1 , get_values_at_times2 , values = list(pr) , times = list(t0) , at = t1 , list = T )

ci_at_times <- get_z2( ci_at_times )

}else{

t1 <- t0

sd_at_times <- sd

ci_at_times <- pr

}

if(conf.int){

sap<-lapply( 1:m , get_est_mi2 , trans = trans , imp_sets = sets , data = data , cens.code = cens.code , r2 = r1 )

#obtain data frame of standard errors and point estimates

cis <- sapply(sap,function(x) x[['est']])

t3 <- sapply(sap,function(x) x[['time']])

#get standard errors and point estimates at single times

cis_at_times<-sapply( 1:m , get_values_at_times2 , values = cis , times = t3 , at = t1 , list = F )

cis_at_times <- get_z2( cis_at_times )

CI <- post_point_est_CI( beta = cis_at_times , sd = sd_at_times , times = t1 , conf.int = conf.int , alpha = alpha )

CI <- unique(replace(CI , is.na(CI) , 0 ))

}else{

CI<-rbind(c(time = 0 , est = 0 ) , data.frame( time = t0 , est = pr ) )

CI <- unique(replace(CI , is.na(CI) , 0 ) )

}

ret<-list( est = CI , call = cl , data = data , cens.code = cens.code , status = status , conf.int = conf.int )

class(ret) <- 'MI_ci'

return(ret)

}

print.MI_ci_1 <- function (x , ... ) {

cat('\nCumulative incidence estimation for interval censored data using multiple imputation\n')

cat( "\nCall:\n", paste( deparse(x$call) , sep = "\n" , collapse = "\n" ) , "\n\n" , sep = "")

cat('Interval-censored response for cumulative incidence estimate :\n\n')

n<-nrow(x$data)

data<-x$data

cens <- x$cens.code

status<-x$status

cat('No.Observation:', n , '\n')

cat('Patern:\n')

stat<-ifelse(data[,status]==cens,'unknown (right-censored)',as.character(data[,status]))

type<-ifelse(data$right==data$left , 'exact' , NA )

type<-ifelse(data$right!=data$left & data$right!=Inf , 'interval-censored' , type )

type<-ifelse(data[,status]==cens , 'right-censored' , type )

print(table('Cause'=stat, type))

cat('\n')

cat('$est\n')

dimest<-paste(dim(x$est)[1] , 'x' , dim(x$est)[2])

cat(paste('A',dimest,'data frame of required estimates\n'))

print(head(x$est))

}

# plot method for MI_ci objects

# @param x A MI_ci object

# @inheritParams plot.MI_surv

plot.MI_ci_1 <- function (x , xlab = 'Time' , ylab = 'Cumulative incidence' , ... )

{

data <- x$est

conf.int <- x$conf.int

plot( data$time , data$est , xlab = xlab , ylab = ylab , type = 's' , ylim = c(0,1) , bty ='l')

if(conf.int){

lines( data$time , data$uci , lty = 2 , type = 's' )

lines( data$time , data$lci , lty = 2 , type = 's' )

}

}

get.set2 <- function( k , data){

data <- data[,c('left','right')]

df1<-data.frame( data , r = runif(nrow(data)))

w <- with( df1 , left + (right - left) * r )

if1<-ifelse(w == Inf , df1$left , w)

return(if1)

}

get_est_mi2<-function( x , status , trans , imp_sets , data = data , cens.code = cens.code , model = c('Cox','FG') , r2 ){

t1 <- imp_sets[ , x ]

fitCI <- survfit( Surv( time = t1 , event = r2 , type = "mstate" ) ~ 1 )

w <- which( fitCI$states == trans )

pr <- fitCI$pstate[ , w ]

sd <- fitCI$std.err[ , w ]

t1 <- fitCI$time

CI <- list(time = t1 , est = pr , sd = sd )

return( CI )

}

get_values_at_times2 <- function( j , values , times , at , list = F , unique = T ){

if(list==F){

sapply( at , function( x ) {

values[ , j ][ tail( which( sort( unique( times[ , j ] ) ) <= x ) , 1 ) ]

}

)

}else{

if(unique){

sapply( at , function( x ) {

values[[j]][ tail( which( sort( ( times[[j]] ) ) <= x ) , 1 ) ]

}

)

}else{

sapply( at , function( x ) {

values[[j]][ tail( which( sort( unique( times[[j]] ) ) <= x ) , 1 ) ]

}

)

}

}

}

MI.ci_2<-function( k , m , data , status , trans , cens.code , conf.int = F , alpha = 0.05 ){

if( !is.numeric(k) ) stop('k must be an integer')

if( !is.numeric(m) ) stop('m must be an integer')

if( !is.data.frame(data) ) stop('data must be a data.frame')

if( !is.logical(conf.int) ) stop('conf.int must be logical')

if( alpha <= 0 | alpha >= 1 ) stop('alpha must be in ] 0 , 1 [')

#if(k <= 1) stop('You may consider the MI.ci function')

cl<-match.call()

prep <- preproc.crreg_2( data = data , m = m , trans = trans , status = status , cens.code = cens.code )

data_int <- prep$data2

data_fix <- prep$data1

or <- prep$or

I <- prep$I

r2 <- as.character( rep( data[ , status ] , m ) )

r2 <- replace( r2 , r2 == cens.code , 0 )

r1 <- as.character( data[ , status ] )

r1 <- replace( r1 , r1 == cens.code , 0 )

#Multiple Imputation

CI <- MI.ci1( m = m , status = status , trans = trans , cens.code = cens.code,

data = data , conf.int = F , alpha = alpha , ntimes = NULL )$est

CI$diff <- c(0 , diff( CI$est ) )

for(i in 1:k){

ss1<-apply(data_int , 1 , function(x ) subset( CI , time >= as.numeric(x['left']) & time <= as.numeric(x['right']) ) )

tk2<-lapply(seq_len(nrow(data_int)) ,function(X) ss1[[X]]$time)

samples<-t( sapply( seq_len(nrow(data_int)) , function(X) {

pk2 <- ss1[[ X ]]$diff

sapply( 1:m , function(x){

if( sum( pk2 ) & length( pk2 ) > 1 ) sample( tk2[[ X ]] , size = 1 , prob = pk2 )

else mean( tk2[[ X ]] ) } ) } ) )

samples2<-rbind(samples,data_fix)[or,]

times<-as.vector(samples2)

ci<-Surv( time = times , event = r2 , type = 'mstate')

fitCI<-survfit( ci ~ 1 , weights = rep( 1 , length( times ) ) / m , conf.type = 'none')

w <- which( fitCI$states == trans )

sd <- fitCI$std.err[ , w ]

pr <- fitCI$pstate[ , w ]

t0 <- fitCI$time

CI<-unique(rbind(c(time = 0 , est = 0 ) , data.frame( time = t0 , est = pr ) ))

CI$diff <- c(0 , diff( CI$est ) )

}

sap<-lapply( 1:m , get_est_mi2 , trans = trans , imp_sets = samples2 , data = data , r2 = r1 )

#obtain data frame of standard errors and point estimates

cis <- sapply(sap,function(x) x[['est']])

t3 <- sapply(sap,function(x) x[['time']])

#get standard errors and point estimates at single times

cis_at_times<-sapply( 1:m , get_values_at_times2 , values = cis , times = t3 , at = t0 , list = is.list(cis) )

cis_at_times <- get_z2( cis_at_times )

#Amended E Curnow

#Next statement is for checking data that are input into post_point_est_CI function - #not required

#ret<-list( est = cis_at_times, sd=sd, times=t0)

#E Curnow: don't return results of Rubin's rules

#CI <- post_point_est_CI( beta = cis_at_times , sd = sd , times = t0 , conf.int = #conf.int , alpha = alpha )

#if(conf.int){

# colnames(CI)<-c('time','prev','sd','uci','lci')

#CI <- unique(replace(CI , is.na(CI) , 0 ))

#}else{

# colnames(CI)<-c('time','prev')

#CI <- unique(replace(CI , is.na(CI) , 0 ))

#}

#ret<-list( est = CI , call = cl , data = data , cens.code = cens.code , status = status , #conf.int = conf.int )

#class(ret) <- 'MI_ci'

#Added E Curnow

return(sap)

}

#Function to apply adapted version of MI.ci

MICI = function(time, type, nimp)

{

#definitions needed for left and right boundary

left=time

right=as.numeric(ifelse(type==0,"inf",time)) #for right-censored data

#apply boundaries for missing times

left=ifelse(is.na(time),0, left)

right=ifelse(is.na(time),100, right)

midata=data.frame(left=left, right=right, status=type)

imp=MI.ci_2(k=5,m=nimp, data=midata, status="status", trans=1, cens.code=0,

conf.int = F, alpha = 0.05)

return(imp)}

1. **Calculating estimates for each imputation in turn and applying Rubin’s rules**
   1. General function for all imputation methods except Delord and Genin’s

Est.calc = function(imp, nimp)

# imp = output from call of ‘mice’ using one of the functions above, nimp = number

# of imputations

# e.g. ests=Est.calc(imp=imp,nimp=5)

{

agvhdcuminc_est=c()

agvhdcuminc_SE=c()

q2_est=c()

q2_SE=c()

for (j in 1:nimp)

{

fit=Cuminc("time","type",data=complete(imp,j))

agvhdcuminc_est[j]=head(fit[fit$time >=100,],1)$CI.1

agvhdcuminc_SE[j]=head(fit[fit$time >=100,],1)$seCI.1

q2=fit[fit$CI.1>=0.5,]

q2_l=fit[fit$CI.1<=0.49,]

q2_u=fit[fit$CI.1>=0.51,]

f_q2=(head(q2_u,1)$CI.1 - tail(q2_l,1)$CI.1)/

(head(q2_u,1)$time - tail(q2_l,1)$time)

q2_est[j]=head(q2,1)$time

q2_SE[j]=head(q2,1)$seCI.1/f_q2

}

q2_results=mean(q2_est)

q2_SE_results=sqrt(mean(q2_SE^2)+((1+1/nimp)*1/(nimp-1)*sum((q2_est-mean(q2_est))^2)))

cuminc_est_results=mean(agvhdcuminc_est)

cuminc_SE_results=sqrt(mean(agvhdcuminc_SE^2)+((1+1/nimp)*1/(nimp-1)*sum((agvhdcuminc_est-mean(agvhdcuminc_est))^2)))

ests=cbind(cuminc_est_results, cuminc_SE_results, q2_results, q2_SE_results)

return(ests)

}

- 1. Function to be used for Delord and Genin’s method

Est.calc.MICI = function(imp, nimp)

# imp = output from call of MICI function, nimp = number of imputations

# e.g.

# ests=Est.calc.MICI(imp=imp,nimp=5)

{

agvhdcuminc_est=c()

agvhdcuminc_SE=c()

q2_est=c()

q2_SE=c()

for (j in 1:nimp)

{

agvhdcuminc_est[j]=head(imp[[j]]$est[imp[[j]]$time>=100],1)

agvhdcuminc_SE[j]=head(imp[[j]]$sd[imp[[j]]$time>=100],1)

q2=imp[[j]]$time[imp[[j]]$est>=0.5]

q2_l=imp[[j]]$time[imp[[j]]$est<=0.49]

q2_u=imp[[j]]$time[imp[[j]]$est>=0.51]

q2_l_ci=imp[[j]]$est[imp[[j]]$est<=0.49]

q2_u_ci=imp[[j]]$est[imp[[j]]$est>=0.51]

f_q2=(head(q2_u_ci,1) - tail(q2_l_ci,1))/

(head(q2_u,1) - tail(q2_l,1))

q2_est[j]=head(q2,1)

q2_SE[j]=head(imp[[j]]$sd[imp[[j]]$est>=0.5],1)/f_q2

}

q2_results=mean(q2_est)

q2_SE_results=sqrt(mean(q2_SE^2)+((1+1/nimp)*1/(nimp-1)*sum((q2_est-mean(q2_est))^2)))

cuminc_est_results=mean(agvhdcuminc_est)

cuminc_SE_results=sqrt(mean(agvhdcuminc_SE^2)+((1+1/nimp)*1/(nimp-1)*sum((agvhdcuminc_est-mean(agvhdcuminc_est))^2)))

ests=cbind(cuminc_est_results, cuminc_SE_results, q2_results, q2_SE_results)

return(ests)

}

1. **Complete case analysis**

CCA = function(time, type)

{

dset=data.frame(time=time, type=type)

fit=Cuminc("time","type",data=dset)

cuminc_est_results=head(fit[fit$time >=100,],1)$CI.1

cuminc_SE_results=head(fit[fit$time >=100,],1)$seCI.1

q2=fit[fit$CI.1>=0.5,]

q2_l=fit[fit$CI.1<=0.49,]

q2_u=fit[fit$CI.1>=0.51,]

f_q2=(head(q2_u,1)$CI.1 - tail(q2_l,1)$CI.1)/

(head(q2_u,1)$time - tail(q2_l,1)$time)

q2_results=head(q2,1)$time

q2_SE_results=head(q2,1)$seCI.1/f_q2

ests=cbind(cuminc_est_results, cuminc_SE_results, q2_results, q2_SE_results)

return(ests)

}

1. **B-spline sieve semiparametric maximum likelihood approach of Bakoyannis *et al.***

#Adaptation of the bssmle_se function from the ‘intccr’ R package

bssmle_se2 <- function(data,nboot) {

#From bssmle_se R code

tmp <- list()

for(k in 1:nboot){

tmp[[k]] <- data[sample(dim(data)[1], replace = TRUE), ]

}

m <- NULL

no.cores <- parallel::detectCores() - 1

clst <- parallel::makeCluster(no.cores)

doParallel::registerDoParallel(clst)

res.bt <- foreach(m = 1:nboot,

.combine = "rbind",

.packages = c("intccr", "splines", "stats", "alabama", "utils")) %dopar% {

pb <- utils::txtProgressBar(title = "Progress bar for the bootstrapping",

min = 0, max = nboot, style = 3)

utils::setTxtProgressBar(pb, m)

#Amended by E Curnow

tmpfit=ciregic(formula=Surv2(v,u,event=c)~1,tmp[[m]],alpha=c(0,0),nboot=0,do.par=FALSE)

tmppfit <- predict(object = tmpfit, covp=1, times = c(1:100))

pars=c(head(tmppfit$t[tmppfit$cif1>=0.5],1),tmppfit$cif1[tmppfit$t==100])

#End of added code

return(pars)

close(pb)

}

parallel::stopCluster(clst)

rownames(res.bt) <- c()

result <- list(numboot = if(is.vector(res.bt)) 1 else nrow(na.omit(res.bt)),

Sigma = if(is.vector(res.bt)) res.bt else var(na.omit(res.bt)))

#End of Bakoyannis code

return(result$Sigma)

}

#Function to calculate estimates

INTCCR = function(time, type)

{

#definitions needed for left and right boundary

v=as.numeric(time-1)

u= as.numeric(ifelse(type==0,"Inf", time))

#apply boundaries for missing times

v= as.numeric(ifelse(is.na(time),0, v))

u= as.numeric(ifelse(is.na(time),100, u))

#Only 1 competing event allowed so recode event type 3

c= as.numeric(ifelse(type==3,2,type))

dset=data.frame(v=v, u=u, c=c)

#Compute the MLE using the Fine and Gray model

fit=ciregic(formula=Surv2(v,u,event=c)~1,data=dset,alpha=c(0,0),nboot=0,do.par=FALSE)

#Calculate estimates

#Predict CI at 100 days

pfit <- predict(object = fit, covp=1, times = c(1:100))

q2_results=head(pfit$t[pfit$cif1>=0.5],1)

cuminc_est_results=pfit$cif1[pfit$t==100]

#Compute variance using non-parametric bootstrap

Sigma=bssmle_se2(data=dset, nboot=50)

q2_SE_results = sqrt(Sigma[1,1])

cuminc_SE_results = sqrt(Sigma[2,2])

ests=cbind(cuminc_est_results, cuminc_SE_results, q2_results, q2_SE_results)

return(ests)

}

1. **Performance measures**

#Calculating theoretical values of cumulative incidence at 100 days and median time

cuminc100_T=pnorm(log(100), mean=3.26,sd=0.71)*0.65

median_T=ceiling(exp(qnorm(p=0.5*500/325, mean=3.26,sd=0.71)))

#Run desired missing data mechanism and missing data method for each simulated dataset

#Initialise results dataset

results = matrix(NA,1000,4)

#colnames(results)=c("cuminc_est_results", "cuminc_SE_results", "q2_results", #"q2_SE_results")

#Run simulation

for (i in 1:1000)

{

sample1=sample500[sample500$sampno==i,]

#Specify missing data mechanism, for example:

sample1$time.miss=MCAR(dset=sample1,time=sample1$agvhd_time,percent=0.1)

#Specify missing data method, for example:

results[i,]=CCA(time=sample1$time.miss, type=sample1$agvhd_status)

#Example using an imputation method

#imp=MICI(time=sample1$time.miss, type=sample1$agvhd_status, nimp=5)

# results[i,]=Est.calc.MICI(imp=imp,nimp=5)

}

# Calculate standardised bias and average model-based SE for estimands of interest

bias_cuminc=mean(results[,1],na.rm=T)-cuminc100_T

empSE_cuminc=sqrt(var(results[,1],na.rm=T))

stand_bias_cuminc=bias_cuminc/empSE_cuminc

stand_bias_cuminc

ModSE_cuminc=sqrt(mean(results[,2]^2))

ModSE_cuminc

bias_q2=mean(results[,3],na.rm=T)-median_T

empSE_q2=sqrt(var(results[,3],na.rm=T))

stand_bias_q2=bias_q2/empSE_q2

stand_bias_q2

ModSE_q2=sqrt(mean(results[,4]^2))

ModSE_q2
